# Optimal Androgen Deprivation Therapy Combined with Proton Beam Therapy for Prostate Cancer: Results from a Multi-Institutional Study of the Japanese Radiation Oncology Study Group

**DOI:** 10.3390/cancers12061690

**Published:** 2020-06-25

**Authors:** Motohiro Murakami, Hitoshi Ishikawa, Shosei Shimizu, Hiromitsu Iwata, Tomoaki Okimoto, Masaru Takagi, Shigeyuki Murayama, Tetsuo Akimoto, Hitoshi Wada, Takeshi Arimura, Yoshitaka Sato, Masahiko Gosho, Katsumasa Nakamura, Hideyuki Sakurai

**Affiliations:** 1 Department of Radiation Oncology, University of Tsukuba, Faculty of Medicine, Tsukuba, Ibaraki 305-8576, Japan; m.moto.carp@gmail.com (M.M.); shimizu@pmrc.tsukuba.ac.jp (S.S.); hsakurai@pmrc.tsukuba.ac.jp (H.S.); 2 QST Hospital, National Institutes for Quantum and Radiological Science and Technology, Inage, Chiba 263-8555, Japan; 3 Department of Radiation Oncology, Nagoya Proton Therapy Center, Nagoya City West Medical Center, Nagoya, Aichi 462-8508, Japan; h-iwa-ncu@nifty.com; 4 Department of Radiology, Hyogo Ion Beam Medical Center, Tatsuno, Hyogo 679-5165, Japan; kiamoto935@gmail.com (T.O.); mtakagidear@gmail.com (M.T.); 5 Department of Radiation Oncology, Sapporo Teishinkai Hospital, Sapporo, Hokkaido 065-0033, Japan; 6 Proton Therapy Division, Shizuoka Cancer Center Hospital, Nagaizumi, Shizuoka 411-8777, Japan; s.murayama@scchr.jp; 7 Division of Radiation Oncology and Particle Therapy, National Cancer Center Hospital East, Kashiwa, Chiba 277-0882, Japan; takimoto@east.ncc.go.jp; 8 Department of Radiation Oncology, Southern TOHOKU Proton Therapy Center, Koriyama, Fukushima 963-8052, Japan; hhktk981@gmail.com; 9 Medipolis Proton Therapy and Research Center, Ibusuki, Kagoshima 891-0304, Japan; arimura-takeshi@medipolis.org; 10 Proton Therapy Center, Fukui Prefectural Hospital, Fukui, Fukui 910-8526, Japan; y-satou-xn@pref.fukui.lg.jp; 11 Department of Biostatistics, Faculty of Medicine, University of Tsukuba, Tsukuba, Ibaraki 305-8576, Japan; mgosho@md.tsukuba.ac.jp; 12 Department of Radiation Oncology, Hamamatsu University School of Medicine, Hamamatsu, Shizuoka 431-3192, Japan; nakam@hama-med.ac.jp

**Keywords:** prostate cancer, androgen deprivation therapy, proton beam therapy, risk classification, radiation therapy

## Abstract

Background: Androgen deprivation therapy (ADT) combined with radiation therapy benefits intermediate- and high-risk prostate cancer (PC) patients. The optimal ADT duration in combination with high-dose proton beam therapy (PBT) remains unknown. Methods: Intermediate- and high-risk PC patients treated with PBT combined with ADT for various durations were analyzed retrospectively. To assess the relationship between ADT and biochemical relapse-free (bRF) rate, Cox proportional hazards models including T stage, prostate specific antigen (PSA) level, Gleason score (GS), and total radiation dose were used. Results: In the intermediate-risk PC patients (*n* = 520), ADT use improved bRF (HR 0.49, 95% CI 0.26–0.93; *p* = 0.029), especially in those with multiple intermediate-risk factors (T2b–2c, PSA 10–20 ng/mL, and GS 7). In the high-risk PC patients (*n* = 555), a longer ADT duration (>6 months) conferred a benefit for bRF (HR 0.54, 95% CI 0.32–0.90; *p* = 0.018), which was most apparent in patients with multiple high-risk factors (T3a–4, PSA > 20 ng/mL, and GS ≥ 8) treated with ADT for ≥21 months. Conclusions: Short-term (≤6 months) ADT is beneficial for intermediate-risk PC patients, but likely unnecessary for those with a single risk factor, whereas ADT for >6 months is necessary for high-risk PC patients and ADT for ≥21 months might be optimal for those with multiple risk factors in combination of high-dose PBT.

## 1. Introduction

Approximately 1,200,000 people develop prostate cancer (PC) annually worldwide, with 350,000 PC-related deaths per year [1]. In Japan, it was estimated that approximately 100,000 new PC patients were diagnosed, and more than 12,000 men died from the disease in 2018 [2]. In recent large-scale comparative studies evaluating PC-specific mortality (PCSM), disease progression, metastasis, and all-cause mortality, the outcomes of external beam radiotherapy were comparable with those of surgery [3]; consequently, radiation therapy (RT) has become an established curative treatment for PC.

Several randomized trials and meta-analyses performed in the 1990s and 2000s showed a significant benefit of androgen deprivation therapy (ADT) combined with RT in terms of biochemical control and overall survival (OS) among patients with intermediate-risk and high-risk PC [4,5,6,7]. Because the relatively low irradiation doses (65–70 Gy) were used in those trials, the optimal duration of ADT to use in combination with high-dose RT remains unknown [8]. Since ADT has negative impacts on bone, lipid, and glucose metabolism and increases the risks of bone fractures, metabolic syndrome, and diabetes mellitus [9,10,11], it is desirable to perform the minimum ADT necessary to control PC. 

Charged-particle RT using protons or heavy ions is useful for treating PC, because the irradiated volume and dose in the rectum and bladder can be decreased compared with X-ray-based therapies [12]. Thus, proton beam therapy (PBT) offer advantageous physical properties in RT for PC because of its reduced toxicity in normal tissues [13,14]. Iwata et al. reported the long-term outcomes of a multi-institutional survey of PBT for PC conducted by the Japanese Radiation Oncology Study Group, in which 520 patients with intermediate-risk and 556 patients with high-risk PC received PBT between January 2008 and December 2011 [15]. In that survey, each institution decided whether or not to apply ADT to each patient; thus, approximately 50% of the intermediate-risk patients and 88% of the high-risk patients received ADT. The duration of ADT ranged widely, with 34% of high-risk patients treated for ≤6 months and 27.6% treated ≥24 months. 

In the present study, we assessed the role of ADT in intermediate- and high-risk PC patients treated with PBT to analyze the appropriate use of ADT.

## 2. Results

### 2.1. Biochemical Relapse

**Figure 1:**
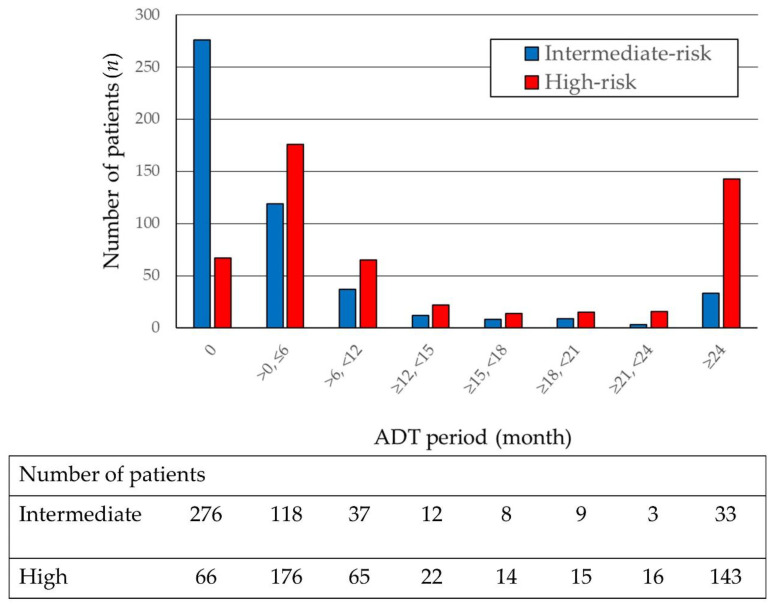
Number of patients according to androgen deprivation therapy (ADT) duration.

The patient and treatment characteristics were reported previously [15]. Of the 1291 patients previously analyzed, 520 had intermediate-risk and 556 had high-risk PC. Table 1 shows the risk factors that determine the National Comprehensive Cancer Network risk classification based on T stage, initial prostate-specific antigen (PSA) levels, and Gleason score (GS) and the numbers of patients with each risk factor. Of the intermediate-risk PC patients, 318 (61.1%) had one risk factor, 170 (32.6%) had two risk factors, and 31 (5.9%) had all three risk factors (T2b–2c, PSA 10–20 ng/mL, and GS 7), with biochemical relapse (bR) observed in 23 (7.2%), 18 (10.6%), and five (16.7%) patients in these groups, respectively. Of the high-risk PC patients, 361 (64.9%) had one risk factor, 129 (23.2%) had two, and 66 (11.8%) had all three high-risk factors (T3a–4, PSA > 20 ng/mL, and GS ≥ 8), with bR observed in 42 (11.6%), 24 (18.6%), and 18 (27.3%) patients, respectively. The durations of ADT in the intermediate-risk and high-risk groups are presented in Figure 1.

### 2.2. Intermediate-Risk PC Group

**Figure 2:**
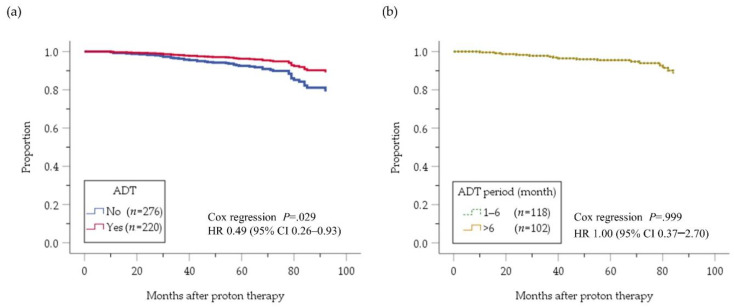
Cumulative bRF survival function adjusted by risk factors of intermediate-risk PC patients; with and without ADT (**a**) and with ADT for 1–6 months and >6 months (**b**). bRF, biochemical relapse-free; ADT, androgen deviation therapy; HR, hazard ratio; CI, confidence interval.

**Figure 3:**
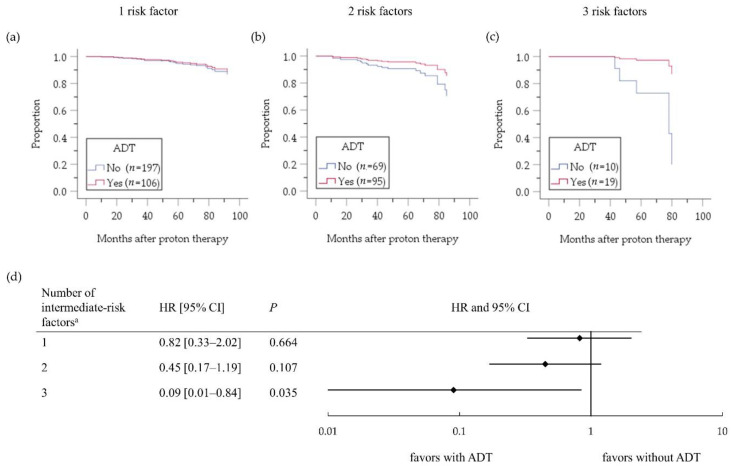
Cumulative survival function according to the number of risk factors; (**a**–**c**) and forest plots (**d**) assessing the risk of biochemical relapse in intermediate-risk PC patients according to the number of intermediate-risk factors ^a^. ADT, androgen deprivation therapy; HR, hazard ratio; CI, confidence interval. ^a^ The three intermediate-risk factors are T stage 2b–2c, PSA level 10–20 ng/mL, and Gleason score 7.

Figure 2 depicts the cumulative biochemical relapse-free (bRF) survival function in the intermediate-risk PC patients who received versus those who did not receive ADT. A benefit of ADT on bRF was evident (hazard ratio (HR) 0.49, 95% confidence interval (CI) 0.26–0.93; *p* = 0.029). A longer ADT duration (>6 months) conferred no survival benefit compared with ADT for ≤6 months (HR 1.00, 95% CI 0.37–2.69; *p* = 0.999); The hazard ratio is very close to 1, and the two curves showing cumulative survival functions adjusted by prognostic factors superimposed each other. Figure 3 depicts a subgroup analysis of the effect of ADT on bRF survival according to the number of factors. The benefit of ADT was more evident in patients with multiple (two or three) intermediate-risk factors compared with a single risk factor. Furthermore, the bRF rate was not improved by ADT for >6 months compared with ≤6 months, even among the patients with multiple intermediate risk factors (HR 1.27, 95% CI 0.29–5.53; *p* = 0.750). 

### 2.3. High-Risk PC Group

**Figure 4:**
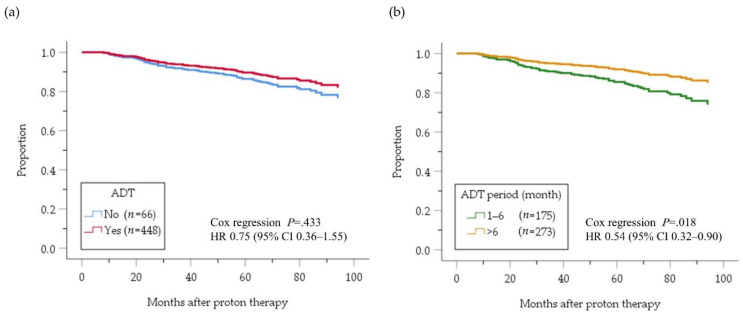
Cumulative bRF survival function adjusted by risk factors of high-risk patients; with and without ADT (**a**) and with ADT for 1–6 months and with ADT >6 months (**b**). The two lines other than “<6” overlap. bRF, biochemical relapse-free; ADT, androgen deviation therapy; HR, hazard ratio; CI, confidence interval. ^a^ Cox regression analysis of the two groups with ADT.

**Figure 5:**
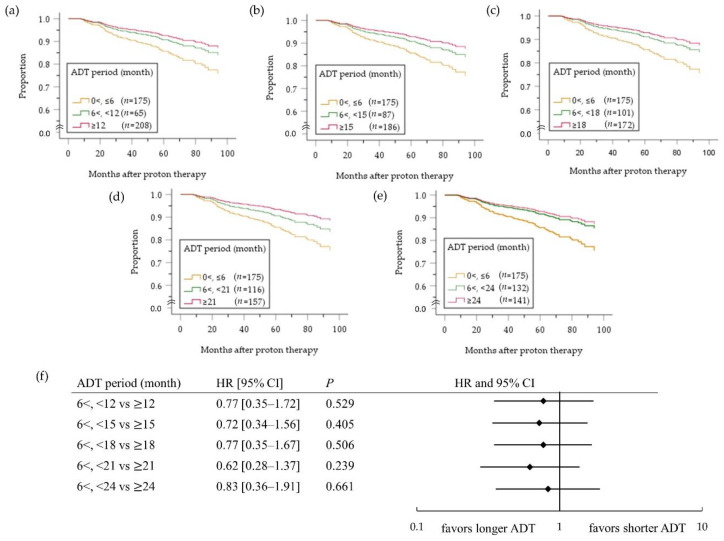
Cumulative survival function (**a**–**e**) and forest plots (**f**) assessing the risk of biochemical relapse in high-risk PC patients with ADT according to the ADT duration (months): <X vs. ≥X months (a: X = 12; b: X = 15; c: X = 18; d: X = 21; e: X = 24 months). ADT, androgen deprivation therapy; HR, hazard ratio; CI, confidence interval.

**Figure 6:**
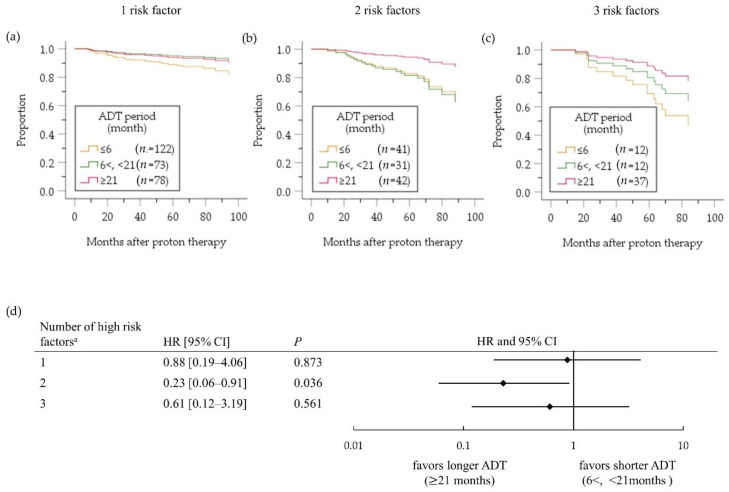
Cumulative survival function (**a**–**c**) and forest plots (**d**) assessing the risk of biochemical relapse in high-risk patients according to the number of high-risk factors ^a^. ADT, androgen deprivation therapy; HR, hazard ratio; CI, confidence interval. ^a^ The three high-risk factors are T stage 3a–4, PSA level >20 ng/mL, and Gleason score ≥8.

**Figure 7:**
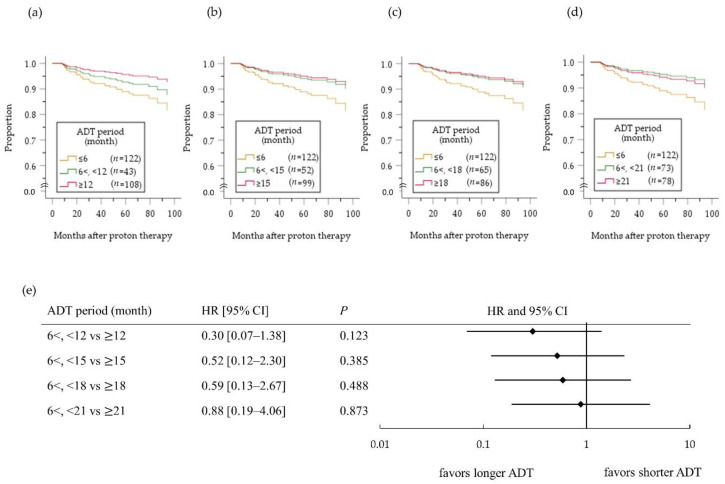
Cumulative survival function (**a**–**d**) and forest plots (**e**) assessing the risk of biochemical relapse in high-risk patients with a single high-risk factor who received ADT according to the ADT duration (months): <X vs. ≥X months (a: X = 12; b: X = 15; c: X = 18; d: X = 21 months). ADT, androgen deprivation therapy; HR, hazard ratio; CI, confidence interval.

Figure 4 depicts the cumulative bRF survival function in the high-risk PC patients. A benefit of ADT on bRF survival of all high-risk patients was masked (HR 0.75, 95%CI 0.36–1.55; *p* = 0.433) by influence of no benefit of ADT for ≤6 months compared with no ADT (HR 1.01, 95%CI 0.47–2.17; *p* = 0.982, Figure 4a). On the other hand, a benefit of ADT for >6 months was evident compared with ADT for ≤6 months (HR 0.54, 95% CI 0.32–0.90; *p* = 0.018, Figure 4b). In an analysis comparing patients treated with ADT for <X months versus ≥X months (Figure 5), the difference between two groups was largest when X was 21 months (Figure 5d), although it did not reach the significance level (HR 0.62, 95% CI 0.28–1.37; *p* = 0.239). In a subgroup analysis according to the number of high-risk factors (Figure 6), however, ADT for ≥21 months improved bRF survival function in 114 patients with two high-risk factors, but not those with one high-risk factor. In the patients with three high-risk factors, a longer ADT duration showed no statistically significant benefit compared with a shorter duration because of small number of patients (*n* = 61), yet tended to be effective for improve survival function of bRF. Among the patients with a single high-risk factor, the difference in survival function for bRF between patients who received ADT for 6 to 12 months compared with ≥12 months was greatest but was not statistically significant (HR 0.30 95% CI 0.07–1.38; *p* = 0.123, Figure 7). 

## 3. Discussion

This study demonstrated that short-term ADT used in combination with PBT has a benefit for improving bRF among patients with intermediate-risk PC, but no additional benefit for a longer ADT duration of >6 months. A combination of ADT for 5–7 months and intensity-modulated RT at ≥81 Gy improved bRF survival, distant metastasis, and PCSM rates in patients with intermediate-risk PC [16]. On the other hand, another study showed that extending the total ADT duration from 16 to 36 weeks before and during RT did not improve the outcomes, including bRF, OS, and PCSM, of patients with intermediate-risk PC [17]. Our study suggests that the results of those previous reports should also apply to PBT. Although it was reported that ADT for <6 months improves event-free survival [18], we cannot discuss whether ADT for 3–4 months is sufficient because almost all patients in the short-term ADT group (≤6 months) received a combination of six-month ADT with PBT. From the viewpoint of therapeutic toxicity, prostate volume reduction by neoadjuvant ADT is desirable. The most significant reduction is expected at three months after ADT initiation [19], and thus the optimal duration is considered to be at least three months.

In the subgroup analysis according to the number of intermediate-risk factors, ADT did not improve the bRF of patients with one risk factor, suggesting no need for ADT in this subgroup. Kawamura et al. reported a five-year bRF survival rate of 97.5% in intermediate-risk PC patients with a T stage of T1c–2b, GS of 7 (3 + 4), and PSA level <10 ng/mL after treatment with high-dose carbon ion radiotherapy alone, and it was not inferior to the corresponding rate of 92.7% in the low-risk group (T stage of T1c–2b, GS≤6, and PSA level <10 ng/mL) [20]. A review by Serrano et al. [21] also indicated that patients with favorable intermediate-risk PC (one intermediate-risk factor: GS ≤7 (3 + 4) or <50% positive biopsy cores) were candidates for active surveillance, dose-escalated RT without ADT, or standard-dose RT plus short-term ADT. It is difficult to determine whether our results are consistent with those previous findings, because we have no information on the GS 3+4 or 4+3 status or number of positive cores. However, it is reasonable to consider that dose-escalated RT alone is sufficient for some patients with intermediate-risk PC. Further studies are needed to clarify the exact patient conditions appropriate for ADT.

In the high-risk PC group, ADT for >6 months, especially ≥21 months was effective for improving bRF. Zapatero et al. performed a randomized trial evaluating high-dose RT with long-term (24 months) compared with short-term (four months) ADT [22] and showed that the long-term ADT improved biochemical control and OS in PC patients, particularly in those with high-risk disease. On the other hand, another randomized trial demonstrated no difference in survival after RT combined with ADT for 36 months compared with 18 months [23]. The results of the current study seem to be consistent with those previous studies of X-ray-based therapies. In addition, a benefit of ADT for ≥21 months was not observed in patients with a single high-risk factor, and shorter ADT durations may be sufficient for these patients. Although the appropriate duration of ADT could not be determined in the present study, the curve of bRF in the use of ADT for ≥12 months tended to be better than that in ADT for more than six but less than 12 months for high-risk PC patients with a single risk factor (Figure 7). The optimal ADT use in combination with high-dose PBT should be determined in future randomized controlled trials.

Because this retrospective study was limited by potential selection bias, lack of randomization, and differences in treatment protocols including total doses and fractionations, especially the use of ADT among institutions, potential differences in these factors were controlled for in our comparisons. In addition, ADT for ≤6 months was roughly six months for all patients. Therefore, it was not possible to compare shorter ADT durations such as three to four months. The lack of significant differences in some of the subanalyses can be due to insufficient sample size in the present study. We performed a subgroup analysis according to the number of risk factors, but it was unclear which factors were particularly important due to the small number of events. However, no differences in the incidence of bR according to the risk factors PSA level, Gleason score, and T stage were observed in intermediate-risk or high-risk PC patients with a single risk factor (Table S1). In addition, an assessment for OS was not performed due to the small number of events, but it has been reported that bR occurring after RT affects OS [24,25]. A multi-institutional prospective clinical trial of PBT for localized intermediate-risk PC in Japan is currently underway and will provide further evidence (UMIN000025453).

## 4. Materials and Methods

The details and methods of the original retrospective survey have been described previously [15]. Briefly, Japanese men with localized PC who received PBT between January 2008 and December 2011 at all seven centers in Japan were enrolled in a multi-institutional retrospective analysis. The current study was approved by the institutional review board and local ethics committee of University of Tsukuba Hospital (document number H30-190). PBT was delivered to the prostate at a total dose of 70–80 GyE in 35–40 fractions (2 GyE/day) or 63–66 GyE in 21–22 fractions (3 GyE/day). We limited our analysis to patients with intermediate- or high-risk PC, as defined by the National Comprehensive Cancer Network; however, the very-high-risk group in the original survey was included within the high-risk group of the present study.

All statistical analyses were performed using SPSS statistical software, version 25 (IBM Corp). Statistical significance was set at *p* < 0.05. We estimated the HRs for bRF using Cox’s proportional hazards models [26]. T stage, PSA level, GS, and total radiation dose were included in the model as covariates. In the intermediate-risk group, patients who received versus did not receive ADT were compared first, and then patients who received ADT for >6 months versus ≤6 months were compared. Among the patients who received versus did not receive ADT, we performed a subgroup analysis according to the number of intermediate-risk factors (T stage 2b–2c, PSA 10–20 ng/mL, and GS 7). In addition, we performed a prospective analysis comparing patients with two risk factors who received ADT for >6 months versus ≤6 months; a similar analysis of the patients with three risk factors could not be performed because of the small number of bR events. Among the patients with one risk factor, those who received versus did not receive ADT were compared according to each risk factor present. 

In the high-risk patients, the following comparisons were performed: with versus without ADT, ADT for ≤6 months versus >6 months, and ADT for <X months versus ≥X months, where X = 12, 15, 18, 21, or 24 months. In the third comparison, for the timepoint at which the largest difference between the two groups was evident, we performed a subgroup analysis according to the number of high-risk factors: T stage 3a–4, PSA level >20 ng/mL, and GS ≥ 8. Similar to the intermediate-risk patients, among the high-risk patients with a single high-risk factor, we compared those with ADT for ≤6 months versus >6 months according to each risk factor present.

## 5. Conclusions

This retrospective analysis of high-dose PBT and ADT for Japanese PC patients showed that short-term adjuvant ADT is necessary for intermediate-risk PC patients with multiple risk factors (i.e., T2b–T2c, PSA level 10–20 ng/mL, or GS 7), but it may be unnecessary for those with a single risk factor. Similarly, although ADT for >6 months should be combined with high-dose PBT for high-risk PC patients, the effect of ADT may be reduced among those with only one risk factor. The appropriate duration of ADT for high-risk PC according to the number of risk factors should be determined in randomized trials, but the present study suggests that ADT for 12 months and 21 months is preferable for those with single and multiple risk factors, respectively, with high-dose PBT.

## Figures and Tables

**Table 1:** Patient characteristics according to the National Comprehensive Cancer Network risk classification.

Variable	Level	Intermediate-Risk PC Group	High-Risk PC Group
Number	*n*	520	555
Age	Mean ± SD	67 ± 7	69 ± 7
T stage	1c–2a	378 (72.7%)	233 (42.2%)
	2b–2c	142 (27.3%)	123 (22.6%)
	3a–4	0	195 (35.2%)
PSA level (ng/mL)	<10	319 (61.6%)	228 (41.1%)
	10–20	199 (38.4%)	112 (20.2%)
	>20	0	215 (38.7%)
Gleason score	5–6	112 (21.5%)	27 (4.9%)
	7	408 (78.5%)	119 (21.6%)
	8–10	0	406 (73.5%)
Number of intermediate-risk factors ^a^	1	318 (61.2%)	
	2	170 (32.8%)	
	3	31 (6.0%)	
Number of	1		360 (64.9%)
high-risk factors ^b^	2		129 (23.2%)
	3		66 (11.9%)

^a^ The intermediate-risk factors are T stage 2b–2c, PSA level 10–20 ng/mL, and Gleason score 7. ^b^ The high-risk factors are T stage 3a–4, PSA level > 20 ng/mL, and Gleason score ≥ 8.

## Supplementary Materials

The following is available online at https://www.mdpi.com/2072-6694/12/6/1690/s1, Table S1: The hazard ratios for biochemical relapse-free rates of intermediate- and high-risk patients according to each risk factor.
