# Supplementary material for: Optimal Androgen Deprivation Therapy Combined with Proton Beam Therapy for Prostate Cancer: Results from a Multi-Institutional Study of the Japanese Radiation Oncology Study Group
Source: Cancers (Basel). 2020 Jun 25;12(6):1690. doi: 10.3390/cancers12061690 (PMC7352923; doi:10.3390/cancers12061690)
Supplement: Supplementary file 1 [file cancers-12-01690-s001.pdf]

cancers

MDPI

Article

Optimal Androgen Deprivation Therapy Combined with Proton Beam Therapy for Prostate Cancer: Results from a Multi-Institutional Study of the Japanese Radiation Oncology Study Group

Motohiro Murakami, Hitoshi Ishikawa, Shosei Shimizu, Hiromitsu Iwata, Tomoaki Okimoto, Masaru Takagi, Shigeyuki Murayama, Tetsuo Akimoto, Hitoshi Wada, Takeshi Arimura, Yoshitaka Sato, Masahiko Gosho, Katsumasa Nakamura and Hideyuki Sakurai

Supplementary Materials:

Table S1. Hazard ratios for biochemical relapse-free survival in intermediate- and high-risk PC patients with a single risk factor.

NCCN risk classification	Risk factor	HR (95% CI)	*p*
	T stage 2b–2c	3.71 (0.19-73.85)	0.391
Intermediate-risk factor [a]	PSA level 10–20 ng/mL	3.84 (0.39-37.67)	0.248
	Gleason score 7	0.42 (0.11–1.52)	0.186
	T stage 3a–4	1.68 (0.14-19.71)	0.681
High-risk factor [b]	PSA level >20 ng/mL	*	*
	Gleason score ≥8	0.48 (0.20–1.14)	0.097

[a] Intermediate-risk patients who received versus did not receive ADT were compared.[b] High-risk patients who received ADT for ≤6 months versus >6 months were compared.* Analysis could not be performed because there were no biochemical relapse events in the patients who received ADT for >6 months.ADT, androgen deprivation therapy; HR, hazard ratio; CI, confidence interval.

© 2020 by the authors. Submitted for possible open access publication under the terms and conditions of the Creative Commons Attribution (CC BY) license (http://creativecommons.org/licenses/by/4.0/).
